# Perfluorooctane Sulfonate (PFOS) and Perfluorohexane Sulfonate (PFHxS) Alters Protein Phosphorylation, Increase ROS Levels and DNA Fragmentation during In Vitro Capacitation of Boar Spermatozoa

**DOI:** 10.3390/ani10101934

**Published:** 2020-10-21

**Authors:** Iván Oseguera-López, Serafín Pérez-Cerezales, Paola Berenice Ortiz-Sánchez, Oscar Mondragon-Payne, Raúl Sánchez-Sánchez, Irma Jiménez-Morales, Reyna Fierro, Humberto González-Márquez

**Affiliations:** 1 Doctorado en Ciencias Biológicas y de la Salud, Universidad Autónoma Metropolitana, Mexico City 09340, Mexico; ivanoslo@yahoo.com.mx (I.O.-L.); biobere.fis@gmail.com (P.B.O.-S.); 2 Departamento de Reproducción Animal, Instituto Nacional de Investigación y Tecnología Agraria y Alimentaria, 28040 Madrid, Spain; perez.serafin@inia.es (S.P.-C.); raulss@inia.es (R.S.-S.); 3 Maestría en Biología Experimental, Universidad Autónoma Metropolitana, Mexico City 09340, Mexico; oscar_payne@hotmail.com; 4 Departamento de Ciencias de la Salud, Universidad Autónoma Metropolitana-Iztapalapa, Mexico City 09340, Mexico; jimi@xanum.uam.mx (I.J.-M.); reyna@xanum.uam.mx (R.F.)

**Keywords:** boar spermatozoa, perfluorinated compounds, PFHxS, PFOS, spermatozoa toxicology

## Abstract

**Simple Summary:**

Perfluorinated compounds are synthetic chemicals, with a wide variety of applications like firefighting foams, food packaging, additives in paper and fabrics to avoid dyes. Perfluorooctane sulfonate and perfluorohexane sulfonate are globally distributed, and contaminates air, water, food, and dust, have toxic effects and bioaccumulate. Significant levels of these compounds have found in blood serum, breast milk, and semen of occupationally exposed and unexposed people, as well as in blood serum and organs of the domestic, farm, and wild animals. The present study seeks to analyze the toxic effects and possible alterations caused by the presence of these compounds in boar sperm during the in vitro capacitation, due to their toxicity, worldwide distribution, and lack of information in spermatozoa physiology during pre-fertilization processes.

**Abstract:**

Perfluorooctane sulfonate (PFOS) and perfluorohexane sulfonate (PFHxS) are toxic and bioaccumulative, included in the Stockholm Convention’s list as persistent organic pollutants. Due to their toxicity, worldwide distribution, and lack of information in spermatozoa physiology during pre-fertilization processes, the present study seeks to analyze the toxic effects and possible alterations caused by the presence of these compounds in boar sperm during the in vitro capacitation. The spermatozoa capacitation was performed in supplemented TALP-Hepes media and mean lethal concentration values of 460.55 μM for PFOS, and 1930.60 μM for PFHxS were obtained. Results by chlortetracycline staining showed that intracellular Ca^2+^ patterns bound to membrane proteins were scarcely affected by PFOS. The spontaneous acrosome reaction determined by FITC-PNA was significantly reduced by PFOS and slightly increased by PFHxS. Both toxic compounds significantly alter the normal capacitation process from 30 min of exposure. An increase in ROS production was observed by flow cytometry and considerable DNA fragmentation by the comet assay. The immunocytochemistry showed a decrease of tyrosine phosphorylation in proteins of the equatorial and acrosomal zone of the spermatozoa head. In conclusion, PFOS and PFHxS have toxic effects on the sperm, causing mortality and altering vital parameters for proper sperm capacitation.

## 1. Introduction

Perfluorinated compounds (PFCs) are characterized by a fully fluorinated hydrophobic linear carbon chain attached to one or more hydrophilic head groups [1]. Due to their properties as hydro-oil repellents and surfactants that are resistant to chemical and biological degradation, as well as their thermal stability [2,3,4], they are used widely in applications and in such products as paints, lubricants, stain repellents, additives for paper products, and aqueous film-forming foams used to fight electrical fires [5,6]. Due to their toxicity, bioaccumulation, and environmental persistence, they appear on the Stockholm Convention’s list of Persistent Organic Pollutants [5,7].

Perfluorooctane sulfonate (PFOS) and perfluorohexane sulfonate (PFHxS) have been detected in human populations worldwide. The highest PFOS and PFHxS levels in blood were reported in retired fluorochemical production workers at 799 ng/mL (range, 14–3,490), and 290 ng/mL (range, 16–1,295), respectively, with calculated half-lives for elimination in serum (arithmetic and geometric mean) of 5.4–4.8, and 8.5–7.3 years, respectively [8]. However, PFCs residues are not present only in humans, as studies have detected them in farm animals like boars, cows, and chickens, among others [9,10]. PFOS in the amounts of ≤1780 and ≤28.6 (μg/kg) were found in liver and muscle samples of domestic and wild boars [11]. Other authors found 0.37 ng/mL in blood samples, and 54 ng/g in liver samples, though the PFHxS residues detected were not significant [9]. The domestic boar is a useful farm animal because of its economic importance. It is also considered an appropriate model for several areas of medical research, such as metabolic and infectious studies [12]. Boars are closely related to humans in terms of anatomy, genetics and physiology, so they are an excellent animal model for study, as genetically modified models can be created for use in modeling human reproduction and pathologies [13,14].

PFCs affect reproductive physiology in several species. In humans, PFOS and PFHxS have been shown to reduce morphologically normal spermatozoa, while PFOS increased spermatozoa tail abnormalities and decreased testosterone levels and motility [15,16,17]. In mice [18], PFOS diminished serum testosterone concentrations and epididymal spermatozoa counts, while in rats it damaged the blood-testis barrier function by disrupting the tight junction-permeability barrier of Sertoli cells [19,20]. In zebra fish, it decreased spermatozoa quality and had an estrogenic effect that increased estradiol levels but decreased testosterone in the juvenile phase [21].

Studies have shown the effects of PFCs on spermatozoa quality and morphology, but their impact on pre-fertilization processes such as capacitation and the acrosome reaction have not been described. Capacitation is essential for the successful binding of the spermatozoa to the oocyte and, therefore, fertilization. This process is characterized by biochemical changes that occur inside the female tract. Upon completing their capacitation, spermatozoa can perform exocytosis from the acrosome, called the acrosome reaction. This can be induced in vitro by chemical and biological agents like zona pellucida proteins, calcium ionophores, glycosaminoglycans, and progesterone [22,23].

Due to the global distribution of PFOS and PFHxS, and the lack of information on their effects on spermatozoa physiology during pre-fertilization processes, the aim of this study was to analyze their possible toxicity and physiological alterations on boar spermatozoa during the in vitro capacitation.

## 2. Materials and Methods

### 2.1. Boar Spermatozoa Samples and Incubation Media

All chemicals were purchased from the Sigma Chemical Company (St. Louis, MO, USA), unless otherwise indicated. All experimental procedures were performed according to institutional and European regulations. Semen samples were obtained from the sperm-enriched fraction of the ejaculates of 8 healthy, fertile and multi-breed boars (Duroc, Pietrain and hybrids Large White × Landrace) between 1 and 4 years of age, using the gloved-hand method. The gelatinous fraction was filtered and the post-sperm fraction was not used. All the samples used were obtained in the months of October to May between 8:30 and 10 a.m. and each pig was given 7 days rest between each sample. All assays were carried out with technical duplicates between 3 and 4 h after sample collection. For the standardization of the techniques and to ensure that there were no individual effects, at least 4 samples were obtained per boar. The samples employed were classified as normozoospermic according to established criteria: viability and motility > 80%, concentration > 200 × 10^6^ spermatozoa/mL, and morphological abnormalities < 15% [24]. To determine the concentration, a 1:1000 dilution of the washed sample was diluted in water, a 10 µL aliquot was placed in a Neubauer chamber. The Eosin-Nigrosin stain, described below was used to determine mortality and morphological abnormalities. Sperm motility was determined subjectively. All samples were observed with an optical microscope Nikon Eclipse E400 (Nikon, Tokyo, Japan) at a magnification of 400×. The data from the initial evaluations of the samples used in this protocol are described in Table 1.

After initial evaluation, the samples were centrifuged at 600× *g* for 5 min. The resulting pellet was rinsed with temperate phosphate-buffered saline (PBS) twice and re-suspended in 1 mL of TALP-HEPES capacitation medium (3.1 mM KCl, 100 mM NaCl, 0.29 mM NaH_2_PO_4_·H_2_O, 10 mM Hepes, 2.5 mM NaHCO_3_, 21.6 mM sodium lactate, 2.1 mM CaCl_2_·2H_2_O, 1.5 mM MgCl_2_·6H_2_O, and 10 µg/mL phenol red as the pH indicator). On the day of analyses, the medium was supplemented with 1 mM of sodium pyruvate and 6 mg/mL of bovine serum albumin fraction V, and then adjusted to pH 7.4. To induce capacitation, an aliquot of 5× 10^6^ washed spermatozoa was transferred to 1 mL of TALP-HEPES and incubated for 4 h at 39 °C in a semi-humid atmosphere, with or without PFCs (concentrations indicated below) [25,26,27].

**Figure 1:**
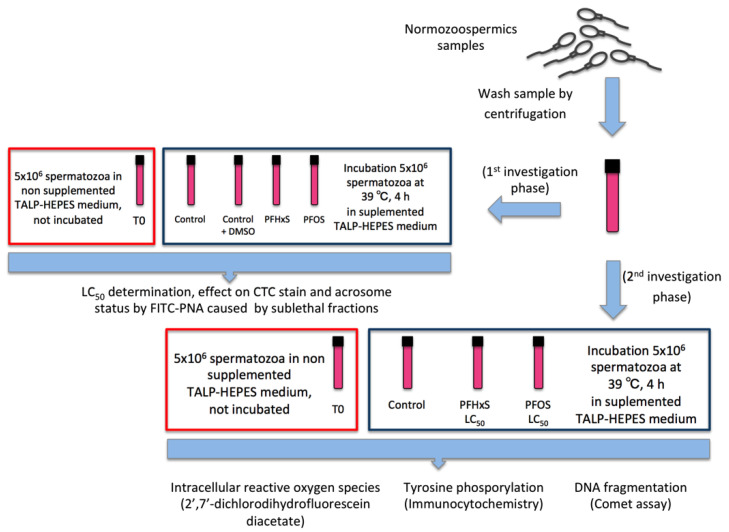
General experimental diagram was designed in two phases. (i) In the first, the LC_50_ values of both PFCs were calculated and the effect of sublethal fractions (⅕ LC_50_, ½ LC_50_, and LC_50_) on sperm capacitation by CTC staining and sAR by FITC-PNA were analyzed. (ii) In the second phase, the effect of the LC_50_ of both PFCs on ROS levels were determined by 2′, 7′-dichlorodihydrofluorescein diacetate, tyrosine phosphorylation by immunocytochemistry and DNA fragmentation with the comet assay.

The spermatozoa experimental groups were:

(1) For the LC_50_ determination.
  - T0, spermatozoa washed and not capacitated.
  - Controls, incubated only in capacitation medium, and to ensure that nontoxic effect of DMSO, used as a diluent of PFCs, 10 µL of DMSO was added in another aliquot.
  - Capacitated in the presence of PFOS (1000, 2000 and 3000 µM) and PFHxS (1000, 2500 and 5000 µM).
(2) For CTC stain and acrosome status by FITC-PNA.
  - T0, spermatozoa washed and not capacitated.
  - Controls, incubated only in capacitation medium, and to ensure that nontoxic effect of DMSO, used as a diluent of PFCs, 10 µL of DMSO was added in another aliquot.
  - Capacitated in the presence of sub-lethal fractions (⅕ LC_50_, ½ LC_50_, LC_50_) of PFOS and PFHxS.
(3) For tyrosine phosphorylation, reactive oxygen species (ROS), and comet assays only LC_50_ concentration of each PFCs were tested. The controls in these last tests were spermatozoa capacitated without the toxics.
(4) Incubation times for experiments above were of 4 h, except in the ROS-determination experiments, where the samples were incubated for 30 and 120 min (Figure 1).
(5) For microscopic studies, at least 200 cells were analyzed per slide.

Live and dead sperm populations were considered for data collection in all the techniques mentioned.

### 2.2. Determination of Mean Lethal Concentration (LC_50_)

The spermatozoa were incubated in capacitation medium supplemented with 1000, 2000 and 3000 µM of PFOS, and 1000, 2500 and 5000 µM of PFHxS under the conditions described previously. The PFCs concentrations used were selected to get a range including the maximal mortality, and thus being able to establish the mean lethal dose. Six ejaculated samples from different boars were used, and were all handled independently. To demonstrate the non-toxicity of the DMSO, the control samples were incubated in the capacitation medium, and in the same medium supplemented with DMSO.

### 2.3. Spermatozoa Viability

Viability was evaluated by eosin-nigrosin staining. A 5 μL drop containing approximately 25,000 spermatozoa was placed on a glass slide temperated at 37 °C, mixed with 5 µL of stain solution (0.67% eosin Y and 10% nigrosin), incubated by 30 s and smeared. Finally, the slide was allowed to air dry at 37 °C. A parallel Hoechst-Propidium Iodide (PI) technique was used. In this case, a 5 µL drop containing approximately 25,000 spermatozoa was placed on a glass slide, mixed with 10 µM of Hoechst, and incubated for 2 min at room temperature (RT). After that, 10 µM of PI were added and the sample was observed immediately under fluorescence microscopy.

### 2.4. Capacitation and the Spontaneous Acrosome Reaction (sAR)

**Figure 2:**
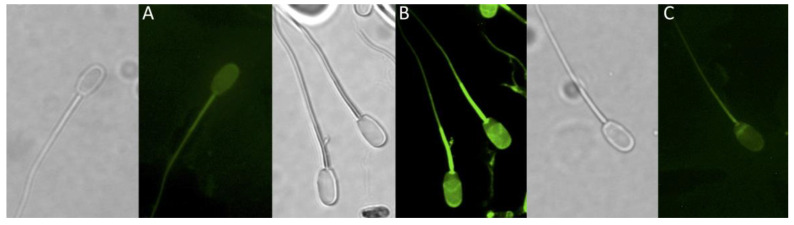
CTC stain patterns. 3 different patters were observed with the use of CTC stain. (**A**) Non-Capacitated, fluorescence throughout the spermatozoa head. (**B**) Capacitated, intense fluorescence in the equatorial and acrosomal zone. (**C**) Acrosomal reacted, fluorescence in equatorial and sometimes in the post-equatorial zone.

The capacitation and sAR processes were measured with ⅕ LC_50_, ½ LC_50_ and LC_50_ of the PFCs. The amounts of non-capacitated spermatozoa (fluorescence throughout the head), capacitated spermatozoa (fluorescence in the acrosome zone), and spermatozoa with sAR (fluorescence in the post-equatorial zone) (Figure 2) were determined by the chlortetracycline (CTC) staining method. A 5 µL drop, containing approximately 25,000 spermatozoa were dropped onto a pre-heated glass slide and mixed with 5 µL of 750 µM of CTC prepared in a buffer containing 20 mM of Tris, 130 mM of NaCl and 5 mM of L-Cysteine. After 30 s, this was mixed with 5 µL of 0.2% glutaraldehyde solubilized in Tris buffer (0.5 mM pH 7.4), mounted with FluoroMount™ (F4680), and gently pressed with a coverslip [28]. Six independent samples were analyzed.

Hoechst-FITC-PNA was used as an alternative method for measuring sAR. For this procedure, a 20-µL sample droplet was placed on a glass slide and air dried at 37 °C. It was then immersed for 30 s in 100% methanol at −20 °C, air dried inside an extraction chamber and stored in a dry atmosphere at RT for later processing. The slide was washed twice for 5 min in PBS, the excess PBS was removed, and 20 µL of 15 µg/mL FITC-PNA prediluted in 5 µg/mL Hoechst were added. This was incubated for 30 min in a wet chamber under darkness, washed for 10 min in double distilled water, dried at 37 °C, and mounted with FluoroMount™ (F4680). Six independent samples were done.

### 2.5. Evaluation of Tyrosine Phosphorylation

**Figure 3:**
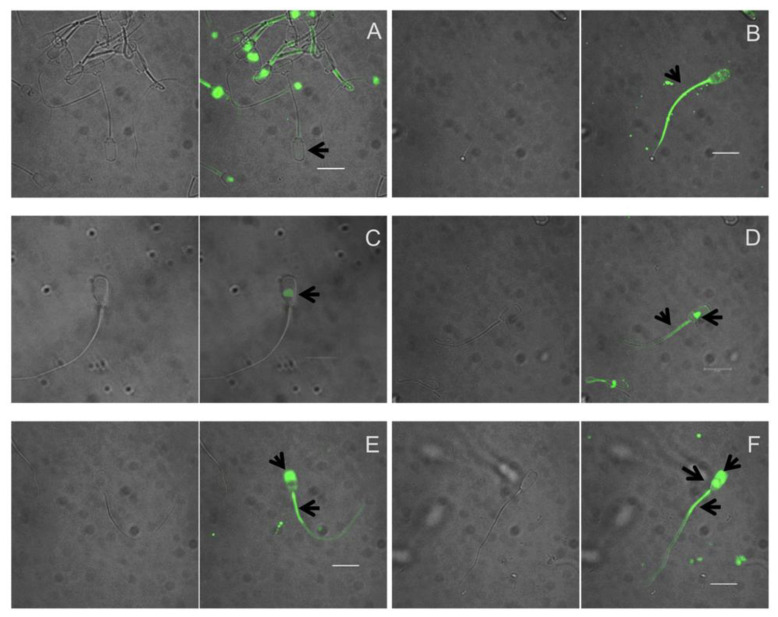
Fluorescence micrographs of various phosphorylation patterns in tyrosine residues of boar sperm capacitated in vitro, the fluorescence was granted by the use of fluorophore Alexa 488. Six different patterns were found, (**A**) No fluorescence. (**B**) Fluorescence in flagellum. (**C**) Fluorescence in equatorial segment. (**D**) Fluorescence in equatorial segment and flagellum. (**E**) Fluorescence in acrosome and flagellum. (**F**) Fluorescence in acrosome, equatorial segment and flagellum. Patterns were classified as: no fluorescence on head (NFH), fluorescence in acrosome (AF), equatorial head segment (EF), and acrosome plus equatorial head segment (AE). —, 1 µm.

To determine tyrosine phosphorylation, immunocytochemistry was performed as described previously with some modifications [29]. Samples of 5 × 10^6^ spermatozoa/mL were washed twice with PBS by centrifugation, then fixed with 2% formaldehyde (Merck, Calbiochem) for 10 min at 37 °C, and permeabilized with 90% methanol (PanReac Quimica, S.A.U., Spain) for 30 min on ice. The samples were rinsed twice with 500 µL of incubation buffer (IB) (PBS 1% BSA), re-suspended in 90 µL of IB, and incubated for 10 min at RT to block them. Next, 5 µL (1:20) of PTyr antibody (anti human-mouse phosphotyrosine, Invitrogen 14-5001-82) were added to each sample and incubated overnight at 4 °C. Samples were rinsed and re-suspended in 95 µL of IB after adding 5 µL (1:20) of secondary antibody (Alexa 488 goat anti-mouse Invitrogen A11029), and incubated for 1 h at RT. Afterwards, they were rinsed with IB by centrifugation and re-suspended in 50 µL of PBS. A 5 µL droplet was placed on a glass slide, mixed with 10 µM of Hoechst to contrast nuclei, and observed under fluorescence microscopy. Three independent samples were done. By fluorescence microscopy a total of 6 phosphorylation patterns were found; no fluorescence and fluorescence in the flagellum, the equatorial segment, the equatorial segment and flagellum, the acrosome and flagellum, the acrosome alone, and the equatorial segment and flagellum (Figure 3). Fluorescence was classified in 4 patterns: no fluorescence in the head (NFH); and fluorescence in the acrosome area (AF), the equatorial head zone (EHZ), and the equatorial + acrosome zone (AE). Images of the patterns observed were captured with a confocal microscope (Leica TCS-SP2, Leica, Germany). To determine fluorescence intensity by flow cytometry, the remaining sample was re-suspended in IB to 500 µL.

### 2.6. Intracellular Reactive Oxygen Species (ROS) Levels

To determine ROS levels, 2′,7′-dichlorodihydrofluorescein diacetate (H2DCFDA) was used, as reported previously, with some modifications [30]. On the day of the experiment, non-capacitated and capacitated spermatozoa were mixed with Hoechst and H2DCFDA to a final concentration of 1 × 10^6^ spermatozoa/mL with 10 µM of Hoechst and 200 μM of H2DCFDA. The spermatozoa were incubated for 30 min at 25 °C. Fluorescence was assessed by flow cytometry. Three independent samples were done.

### 2.7. Single-Cell Gel Electrophoresis (SCGE), or the Comet Assay

The alkaline version of the SCGE was used to measure DNA fragmentation, as described previously [31]. A suspension of 85 µL of 0.5% low-melting point (LMP) agarose in PBS containing 20,000 spermatozoa was placed on a slide previously covered with 1% agarose and covered with a 22 × 22 mm coverslip. For gelation of the LMP agarose, the slides were left overnight in a wet box at 4 °C. After removing the coverslips, the slides were incubated at 37 °C for 1 h in lysis solution (2 M NaCl, 55 mM EDTA-Na2, 8 mM Tris, 4% Triton X-100, 0.1% SDS, 1 mM DTT, and 0.5 mg/mL of proteinase K, pH 8). Next, they were washed twice in alkaline (0.3 M NaOH, 1 mM EDTA-Na2, pH 12) electrophoresis solution, placed in the electrophoresis cell, and filled with the same solution to cover them with about 1 cm of solution. Electrophoresis was run for 10 min at 25 V. The slides were then neutralized in 0.4 M of Tris-HCl pH 7.5 for 10 min. After that, they were fixed in methanol for 3 min and left to air dry prior to staining with ethidium bromide for observation under fluorescence microscopy at 400×. The comets were digitalized with a Nikon 5100 digital camera (Nikon, Japan) coupled to the microscope in manual mode, with a constant configuration. At least 150 comet images were analyzed using the free software, Casplab 1.2.3 beta2 (CaspLab.com) [32]. Four independent samples were done.

### 2.8. Flow Cytometry

For the immunofluorescence assay of tyrosine phosphorylation and the fluorescent probe for intracellular ROS, mean fluorescence intensity (FI) was determined by flow cytometry in a FACSCanto II flow cytometer (BD Biosciences, San Jose, CA, USA). The spermatozoa were gated in the FSC/SSC dot plot to exclude debris, and then confirmed by analyzing nuclear staining with Hoechst using a 405 nm laser and blue filter (450/50 nm). A sample of 5 × 10^6^ spermatozoa was transferred to a BD Falcon 5 mL, round-bottom tube and placed in the flow cytometer at a flow rate of 10 µL/min to count a total of 1 × 10^4^ spermatozoa per determination. For flow cytometer compensation, control samples with only one fluorochrome were prepared with Hoechst for nuclei staining, H2DCFDA for ROS determination, and Alexa 488 for the secondary antibody, in order to determine tyrosine phosphorylation. For both FI analyses, a 488 nm laser and green filter (530/30 nm) were used. The data acquired were analyzed by logarithmic representation using FlowJo software (Becton–Dickinson, USA). Three independent samples were analyzed for each study. Trained personnel performed equipment calibration using the Long Clean mode as per the manufacturer’s instructions. Six independent samples were done.

### 2.9. Fluorescence Microscopy

All samples analyzed by fluorescence microscopy were observed in a Nikon Optiphot-2 microscope (Nikon, Tokyo, Japan). The B-2A filter was used for the CTC, FITC-PNA, and Alexa 488 procedures with the secondary antibody in immunocytochemistry, while the UV-2A filter was utilized for fluorescence emitted by Hoechst. The G-2A filter was employed for the viability test with propidium iodide and the comet assay with ethidium bromide.

### 2.10. Statistical Analyses

The results are expressed as mean ± SD. The Probit test [33] was applied to determine the LC_50_. To establish differences on the viability, capacitation process, AR, SCGE, as well as tyrosine phosphorylation adhesion, the data were subjected to one-way analysis of variance (ANOVA). Two-way ANOVA was used for the statistical analysis of the effect on ROS production. Mean pairwise comparisons were computed with a Tukey’s HSD test with a *p* value ≤ 0.05. Statistical analysis was performed with the IBM SPSS software (IBM SPSS Statistics, version 20 for Mac Os).

## 3. Results

### 3.1. Determination of the LC_50_ of PFOS and PFHxS

**Figure 4:**
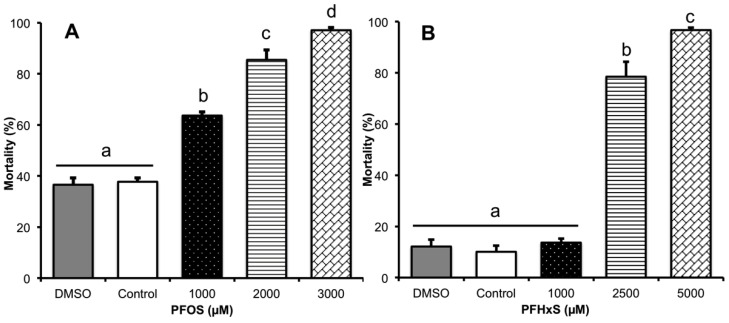
Mortality rates during boar spermatozoa in vitro capacitation process. (**A**) PFOS mortality increases significantly at all experimental concentrations evaluated. (**B**) PFHxS mortality is not significant at a concentration of 1000 µM, but it increases significantly at concentrations of 2500 and 5000 µM. Controls tested do not showed significant differences between them. Mortality data plotted correspond to eosin-nigrosin stain. All spermatozoa stained in pink by eosin (eosin positive) was considered dead, *n* = 6. ^a, b, c, d^ Different lowercase letters indicate the existence of significant differences between treatments (*p* < 0.05).

No significant difference was observed between controls. PFOS showed more spermatozoa toxicity than PFHxS, as 80% mortality was obtained with 1000 μM of PFOS, while 2500 μM were required for PFHxS (Figure 4). The LC_50_ of both compounds was calculated by Probit at 460.55 μM for PFOS and 1930.60 μM for PFHxS.

### 3.2. Effect of Sub-Lethal Fractions on Boar Spermatozoa Capacitation

**Figure 5:**
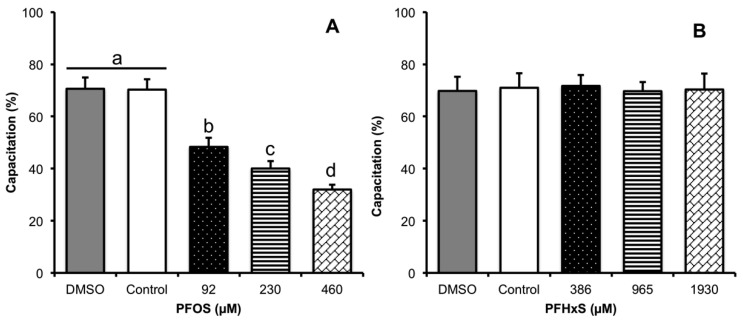
Effect of PFCs on sperm capacitation process. (**A**) The presence of PFOS significantly decreases the percentage of capacitated sperm at all sublethal concentrations tested. No alteration was observed in the sperm capacitation process in the presence of PFHxS as determined by the CTC technique, *n* = 6 (**B**). ^a, b, c, d^ Different lowercase letters indicate the existence of significant differences between treatments (*p* < 0.05). Sperm considered capacitated were CTC positive with pattern B (Figure 2). The sub-lethal concentrations used were ⅕ LC_50_ (92 µM for PFOS and 386 µM for PFHxS), ½ LC_50_ (230 µM for PFOS and 965 µM for PFHxS) and LC_50_ (460 µM for PFOS and 1930 µM for PFHxS).

The sub-lethal concentrations used were ⅕ LC_50_ (92 µM for PFOS and 386 µM for PFHxS), ½ LC_50_ (230 µM for PFOS and 965 µM for PFHxS) and LC_50_ (460 µM for PFOS and 1930 µM for PFHxS). We considered spermatozoa capacitated when they showed fluorescence in the acrosome. PFOS significantly decreased spermatozoa capacitation, from 70.28% ± 4.07 of capacitated spermatozoa in the control to 48.32 ± 3.43% in ⅕ LC_50_, (−22%, *p* < 0.001), 40.03 ± 2.82% in ½ LC_50_ (−30%, *p* < 0.001), and 31.97 ± 1.83% in LC_50_ (−38%, *p* < 0.001). There was no effect upon adding DMSO to the TALP-HEPES medium, as this produced 70.61 ± 4.37% of spermatozoa capacitation (Figure 5A). PFHxS showed no effect on spermatozoa capacitation, determined by the fluorescence pattern of chlortetracycline (Figure 5B), since 69.7–71.6% of the spermatozoa were capacitated.

### 3.3. Subsection Effect of Sub-Lethal Concentrations of PFCs on the sAR Process

**Figure 6:**
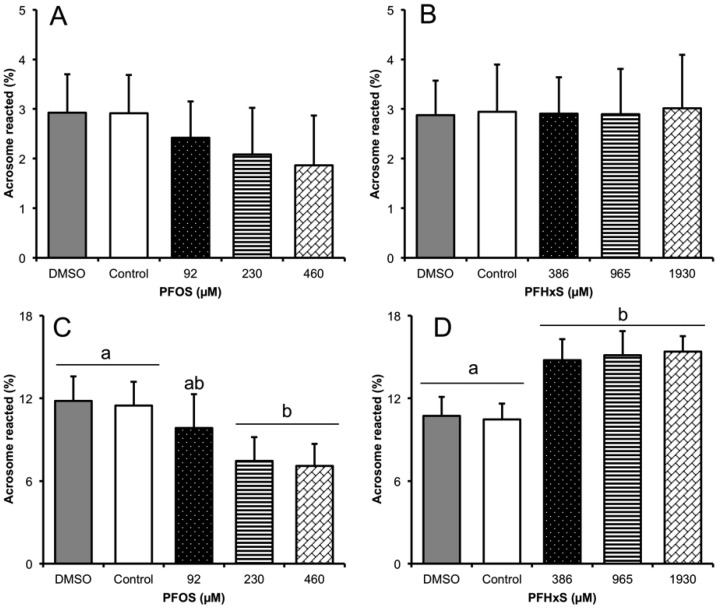
Effect of PFCs on spontaneous acrosome reaction (sAR) *n* = 6. (**A**,**B**), determined by CTC; (**C**,**D**), by FITC-PNA. (**A**) PFOS shows a tendency to decrease sAR, but it is not significant in any concentration. (**B**) PFHxS does not affect sAR in any of the concentrations analyzed. (**C**) PFOS shows a significant decrease from ½ LC_50_, the ⅕ LC_50_ does not differ significantly from the control. (**D**) PFHxS, significantly increases the acrosome reacted in all conditions tested, there was no significant difference between controls. ^a, b^ Different lowercase letters indicate significant differences between treatments (*p* < 0.05).

The sAR process, evaluated by CTC, showed no significant differences at any of the sub-lethal concentrations of PFOS and PFHxS (Figure 6A,B), so we used a PNA lectin-binding assay to further test the effect of PFCs on acrosome status and integrity. The FITC-PNA technique found that PFOS significantly reduced sAR from 11.47 ± 1.74% in the control to 7.45 ± 1.73% (−4.02%, *p* = 0.005) in ½ LC_50_, and 7.09 ± 1.59% (−4.38% *p* = 0.001) in LC_50_. No significant differences were seen in ⅕ LC_50_ compared to controls (Figure 6C). PFHxS, in contrast, significantly increased sAR in ⅕ LC_50_, from 10.46 ± 1.14% in the control to 14.77 ± 1.52% (4.31% *p* = 0.001), and to 15.12 ± 1.75% (4.66% *p* < 0.001) in ½ LC_50_, and 15.39 ± 1.13% (4.93% *p* < 0.001) in LC_50_ (Figure 6D).

### 3.4. Tyrosine Phosphorylation Determination

Tyrosine phosphorylation was evaluated by two mechanisms; first, FI was assessed by flow cytometry, then the fluorescence pattern was measured by fluorescence microscopy.

#### 3.4.1. Flow Cytometry

**Figure 7:**
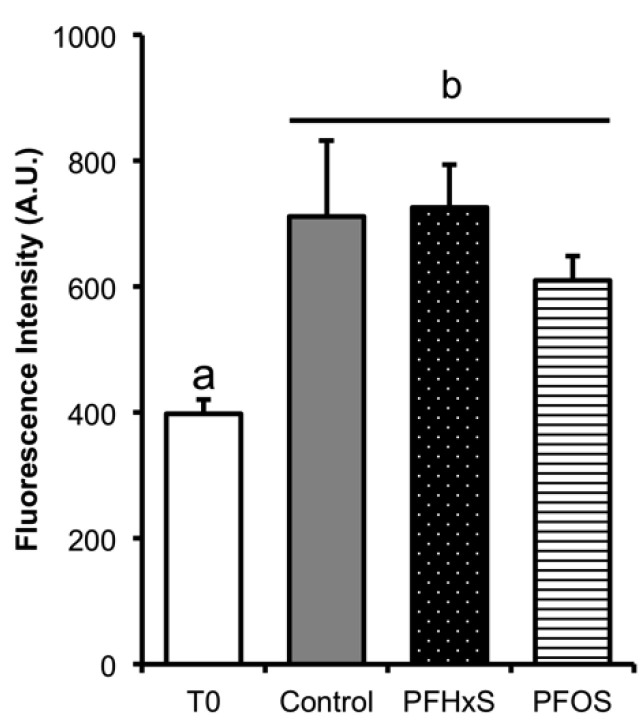
Flow cytometry of tyrosine phosphorylation in boar sperm. The fluorescence intensity obtained by fluorophore Alexa 488 were determined as arbitrary units (AU) did not show significant differences in any of the capacitation conditions tested. A significant difference was observed with the control at time T0, *n* = 3. ^a, b^ Different lowercase letters indicate the existence of significant differences between treatments (*p* < 0.05).

The phosphorylation of tyrosine residues in spermatozoa proteins after capacitation in the absence of PFCs was significantly higher (control = 711.67 ± 120.02 FI) than before capacitation (T0 = 398 ± 23.30 FI) (*p* = 0.003). No significant differences were observed in the samples capacitated in the presence of PFCs, as results were 726 ± 67.45 FI and 610 ± 38.57 FI in LC_50_ of PFHxS and PFOS, respectively (Figure 7).

#### 3.4.2. Fluorescence Microscopy

The NFH pattern was present at T0 (before capacitation) in 9.51 ± 1.03% of the spermatozoa. After capacitation, there were no significant differences in the presence of this pattern in the TALP-HEPES control (12.15 ± 0.80%). After incubation with PFOS LC_50_, a significant increase was observed (12.69 ± 1.50%, *p* = 0.025) compared to T0, though no differences were observed for the other PFOS concentrations or PFHxS.

The AF pattern in T0 was 14.42 ± 0.75%, but after capacitation the control decreased significantly to 5.24 ± 1.8% (*p* < 0.001). The spermatozoa capacitated with PFOS at LC_50_ diminished 4.5 times to 3 ± 1.46% (*p* < 0.001), while with PFHxS at LC_50_ the decrease was 14-fold, to 1.28 ± 1.29% (*p* < 0.001).

EHZ was the most abundant pattern in all conditions, obtaining 75.62 ± 0.98% in T0. This pattern decreased significantly in the spermatozoa capacitated under the control conditions, to 65.38 ± 1.98% (*p* < 0.001). Compared to the control, exposure to LC_50_ of PFOS or PFHxS was significantly higher with results of 71.06 ± 2.50% (*p* = 0.027) and 81.6 ± 1.87% (*p* < 0.001), respectively.

The AE pattern significantly increased after capacitation, from 0.45 ± 0.79 in T0 to 17.23 ± 0.74 in the control (*p* < 0.001). Exposure to LC_50_ of PFOS or PFHxS, however, significantly decreased this AE pattern compared to the control to 13.26 ± 0.73 (*p* = 0.003), and 7.32 ± 1.25 (*p* < 0.001), respectively (Table 1). These results seem to indicate that PFCs slow the capacitation process by modifying the phosphorylation of tyrosine residues (Table 2).

### 3.5. Effect of PFCs on ROS Production

**Figure 8:**
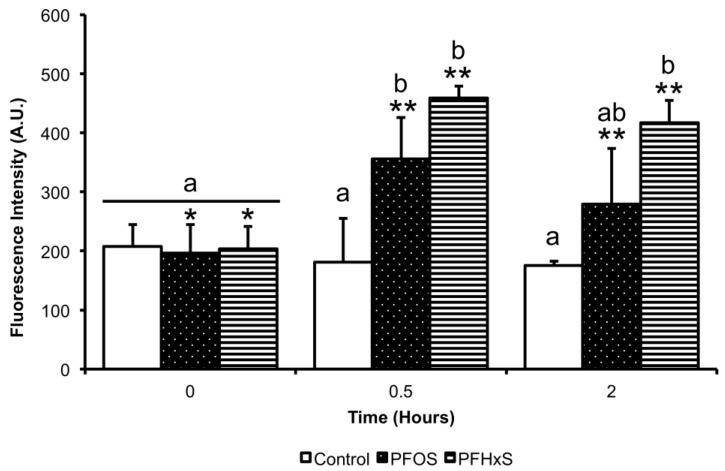
Determination of intracellular ROS by the use of 2′,7′-dichlorodihydrofluorescein diacetate, as a result of exposure to PFCs, *n* = 3. An increase in the fluorescence intensity (AU) of ROS was observed during the first 0.5 h of exposure to PFCs. At 2 h exposure, a decrease in ROS fluorescence intensity was recorded, but still significantly higher on PFHxS treatment, compared to the control. Both, PFOS and PFHxS shows significant differences between same treatment at different times. ^a, b^ Different lowercase letters indicate the existence of significant differences between treatments at same time evaluated (*p* < 0.05). Different asterisk numbers indicate significant differences between the sametreatment at different times evaluated (*p* < 0.05).

The LC_50_ of PFOS and PFHxS caused an over-production of ROS at 30 min of treatment, generating them at 1.8 and 2.3 times, respectively, to 355.67 ± 70.55 (*p* = 0.028) and 459 ± 20.00 arbitrary fluorescence intensity units (A.U.) (*p* = 0.003), compared to the control at 181 ± 74.51. After 2 h of incubation in the capacitation medium, the LC_50_ of PFHxS showed significant differences compared to the control (*p* = 0.006). Upon comparing the same condition at different times, we found that the LC_50_ of PFOS and PFHxS showed significant differences, with values for PFOS of 196.67 ± 47.65 at T0, 355.67 ± 70.55 at 30 min (*p* < 0.001) and 279 ± 94.73 at 2 h (*p* = 0.002). In the case of PFHxS, 203.33 ± 38.19 at T0, 459 ± 20 at 30 min (*p* < 0.001), and 417 ± 37.72 at 2 h (*p* = 0.001) (Figure 8).

### 3.6. Effect of PFCs on DNA Fragmentation

**Figure 9:**
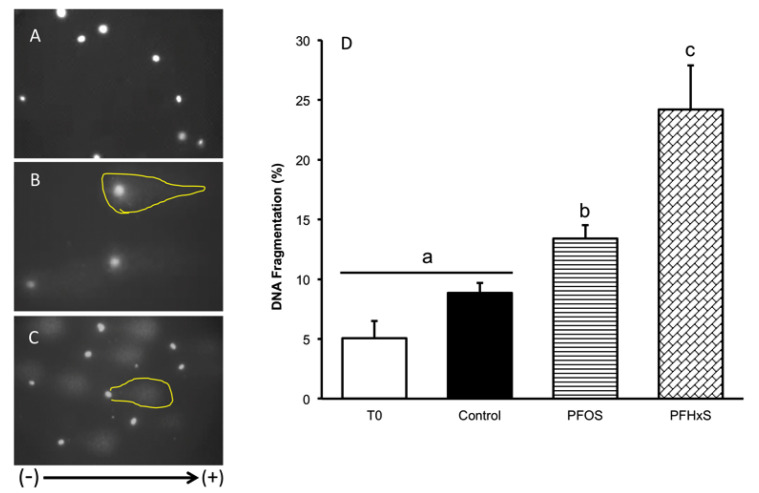
Determination of DNA fragmentation by comet assay, *n* = 4. (**A**) Sample in T0 with low level of fragmentation. (**B**) Sample with medium level of fragmentation after exposure to PFOS, and (**C**) Sample with high level of fragmentation due to exposure to PFHxS. The arrow indicates the direction of migration of DNA fragments to the positive pole, allowing the formation of the comet, the ethidium bromide allows the observation by fluorescence microscopy, yellow line indicates the tail comet area. The graph in (**D**) shows a significant increase in DNA fragmentation, about five and three times, after exposure to PFHxS and PFOS respectively compared to T0. No significant differences between T0 and the Control were detected. ^a, b, c^ Different lowercase letters indicate the existence of significant differences between treatments (*p* < 0.05).

Because the PFCs studied increased ROS production after 30 min, we decided to determine whether this increase generated sperm DNA fragmentation (Figure 9). However, only 5.07 ± 1.42% of the spermatozoa in T0 showed fragmentation, and no significant differences were found in relation to the capacitation control, though there were significant increases, to 13.40 ± 1.12 (*p* = 0.001) with PFOS and 24.2 ± 3.69 (*p* < 0.001) with PFHxS.

## 4. Discussion

Although, many studies have evaluated the effect of PFCs on reproduction, most are of the in vivo type, or involved exposed persons. Very few have evaluated the toxic mechanisms of PFCs. Louis et al. (2015) [34] evaluated the relation between seven PFCs and the parameters of human semen quality, finding poor quality with six of the PFCs evaluated, in the form of abnormal heads, coiled and double tails, and more immature spermatozoa. Their results agree with those from Joensen et al. (2009) [15], who concluded that the combination of PFOA and PFOS decreased the amount of morphologically normal spermatozoa. The toxic effect of PFCs has not been extensively studied, especially not in relation to the physiology of spermatozoa capacitation. For this reason, and to discuss the mechanisms that are altered by the compounds analyzed, we compared the effects of PFCs studied in other cellular and animal models. The present experiment evaluated the effect of PFOS and PFHxS on the capacitation and sAR of boar spermatozoa in vitro. It seems that these two perfluorinated compounds analyzed are not as toxic as expected, as their LC_50_ is in the range of 0.5–2 mM, though this result may be attributable to the fact that the spermatozoa were incubated in the PFCs for only 30–240 min.

Slotkin et al. (2008) [35] evaluated the in vitro neurotoxicity of PFOS, PFOA (perfluorooctanoic acid), PFOSA (perfluorooctane sulfonamide) and PFBS (perfluorobutane sulfonate) in undifferentiated and differentiated PC12 cells. They concluded that each PFC exerted a distinct effect on those cells. PFOSA had the strongest effect, followed by PFOS, PFBS, and finally, PFOA. PFOSA was found to decrease DNA synthesis and cell viability, and to enhance oxidative stress. The authors proposed that the toxic effect of PFOSA could be associated with its greater hydrophobicity, which allowed it to easily access the cell membrane. In the boar spermatozoa analyzed in our study, we observed that PFOS is more toxic than PFHxS, a finding that correlates with its greater hydrophobicity.

The in vitro boar sperm capacitation methodology gave optimal results, obtaining levels of capacitation, similar to others previously reported [27,36]. The CTC technique is useful for differentiating among non-capacitated, capacitated, and AR spermatozoa in normal in vitro sperm, based on the CTC fluorescence patterns when they are in contact with intracellular Ca^2+^ bound to membrane proteins [37]. The results of this assay were also unexpected in light of the literature, for the Ca^2+^ patterns were scarcely affected by PFOS and not at all by PFHxS. Moreover, no changes were observed in sAR with either PFC. A study conducted with hBMSCs cells showed that PFHxS increased calcium transport [38], and that PFOS had a negative effect on suppressed synaptogenesis and inhibited neurite growth caused by abnormal regulation of calcium in the hippocampus [39]. As that work demonstrates, both compounds modify calcium regulation, and likely damage Ca^2+^ channels or Ca^2+^ internal redistribution, as observed in our study model, where exposure to PFHxS had a more evident effect. As all these results show, PFCs alter the transport and distribution of calcium in different cell models. We suspected that this could also affect the ion in spermatozoa, so we decided to use FITC-PNA to determine the status of the acrosome of our samples. As is well-known, the acrosome is a large secretory vesicle biochemically similar to a lysosome, and forms as a product of the Golgi apparatus [40]. It contains two membranes, one internal the other external, and one principle component is the sugar galactosyl β-1,3 N-acetyl galactosamine, which binds specifically to the peanut agglutinin conjugated with fluorescein isothiocyanate (FITC-PNA) that allows observations of acrosome integrity [41,42]. Using this method, we observed opposite effects of PFOS and PFHxS, as the spermatozoa with sAR were seen to decrease significantly when exposed to ½ CL_50_ of PFOS, but exposure to ⅕ of the LC_50_ of PFHxS increased this significantly. As mentioned above, PFOS is more toxic than PFHxS and may kill spermatozoa before they can perform the acrosome reaction. PFHxS in contrast, might take longer to kill cells, thus allowing them to complete sAR.

Boar spermatozoa are divided into 2 parts, the head and the flagellum. The latter is further divided into middle, principle and terminal sections. Signals received in the plasma membrane trigger activation of the signaling cascades for the hyperactivation of mobility. The tyrosine phosphorylation is essential for fertilizing the oocyte, since it is involved in spermatozoa hypermotility, the acrosome reaction, and gamete fusion [43,44].

Our analysis of tyrosine phosphorylation by flow cytometry did not produce significant differences under any conditions tested. However, analyses by fluorescence microscopy did reveal significant differences in proteins in the acrosome and equatorial zone of the spermatozoa head when incubated with the LC_50_ of PFHxS. In addition, incubation with the LC_50_ of PFOS and PFHxS caused significant inhibition in the pattern of full tyrosine phosphorylation (acrosome zone + head equatorial zone). These results seem to suggest that PFCs slow down the capacitation process by modifying tyrosine phosphorylation and inhibiting the change of EHZ and AZ to the AE pattern. This effect of PFCs on spermatozoa tyrosine phosphorylation has not been reported previously. Gao et al. (2017) [20] determined that PFOS damages the Sertoli cells by disturbing actin cytoskeleton by down-regulating p-Akt1-S473 and p-Akt2-S474. They added that SC79 (an Akt1/2 activator) blocked PFOS-induced Sertoli cell injuries by rescuing PFOS-induced F-actin disorganization. Qiu et al. (2016) [45] proposed that the target of PFOS is p38/ATF2, and that this is associated with an increase in phosphorylation in a dose- and time-dependent manner related to perturbations of the blood-testis barrier.

The effect of PFCs on ROS production and its cellular effect have been studied in, for example, SH-SY5Y neuroblast cells, where 25 μM of PFOS significantly generated ROS and caused neurotoxic effects [46]. In liver cells, PFOS caused cytotoxicity associated with ROS and lipid peroxidation with depletion of hepatocyte glutathione. Antioxidants and ROS scavengers inhibited this cytotoxicity [47]. PFHxS had apoptotic effects on cerebellar granule cells and increased the activation of ERK1/2, JNK, and p38 MAPK, but antioxidant treatment blocked these effects [48].

ROS are involved in regulating spermatozoa processes, as observations have shown that an increase in the concentration of these compounds can enhance spermatozoa capacitation, while adding antioxidant substances reduces capacitation [49]. In our model, both compounds significantly increased ROS after 30 min of exposure compared to controls. At 2 h of exposure, ROS decreased, but when analyzed with the LC_50_ of PFHxS the level was still significantly higher than in the control. Despite this increase, the boar spermatozoa exposed to both PFCs did not significantly increase in vitro spermatozoa capacitation. Although, ROS are necessary for sperm functions, some authors have shown that boar sperm is extremely sensitive to these compounds, and have related this to alterations in motility, acrosome integrity, and lipid peroxidation [50]. It may be that damage to the mitochondrial membrane by PFCs increases intracellular ROS levels and affects the membrane, proteins and sperm DNA [51].

As we observed, ROS production was considerable with both compounds. Studies have demonstrated that high ROS concentrations can cause DNA damage [52]. In the present case, we observed significant damage caused by exposure to both compounds, as PFHxS showed approximately 20%, and PFOS 10%, compared to controls, which presented only around 5%. Genomic damage may, therefore, be related to the increased production of ROS caused by the PFCs analyzed herein.

## 5. Conclusions

Perfluorinated compounds appear to damage sperm through different metabolic pathways. It is well known that increased ROS seems to be related to male infertility, and as mentioned above, boar sperm is susceptible to these compounds. Besides, the necessary processes for sperm capacitation, such as tyrosine phosphorylation, are strongly related to an optimal ROS concentration, so an alteration in this balance leads to damage or changes. Therefore, the alterations caused by perfluorinated in this process could cause a lack of sperm-oocyte recognition and prevent fertilization. Furthermore, the increase in ROS significantly damages DNA. The mobilization of intracellular calcium is another possible route of damage. PFHxS exposure affected the normal calcium patterns distribution, so it was impossible to determine capacitation and sAR using the CTC technique.

## Figures and Tables

**Table 1:** Initial sperm evaluation data.

Samples at T0	Assay (%)				
	Eosin-Nigrosin		CTC Stain		FITC-PNA
Variable	Mortality	Abnormalities	Capacitated	sAR	sAR
*n* = 6	5.69 ± 1.64	1.86 ± 0.38	6 ± 1.33	1.86 ± 0.56	2.31 ± 0.33

sAR = Spontaneous Acrosome Reaction. All samples used were classified as normozoospermic according previously mentioned criteria.

**Table 2:** Effect of PCFs in tyrosine residues phosphorylation patterns in boar sperm capacitation.

Treatment	Tyrosine Phosphorylation Patterns															
	NFH				AF				EHZ				AE			
T0	9.51	±	1.03	^a^	14.42	±	0.75	^a^	75.62	±	0.98	^a^	0.45	±	0.79	^a^
Control	12.15	±	0.80	^ab^	5.24	±	1.48	^bc^	65.38	±	1.98	^b^	17.23	±	0.74	^b^
PFOS	12.69	±	1.50	^b^	3.00	±	1.46	^bc^	71.06	±	2.50	^a^	13.26	±	0.73	^c^
PFHxS	9.81	±	0.69	^a^	1.28	±	1.29	^c^	81.6	±	1.87	^c^	7.32	±	1.25	^d^

Sperm capacitation was performed in Talp-Hepes medium at 39 °C for 4 h. Both compounds used in LC_50_, for PFOS 460.55 µM and PFHxS 1930.60 µM. Treatments: T0, non-capacitated sperm sample; Control, capacitated; PFOS, Perfluorooctane Sulfonate y PFHxS, Perfluorohexane Sulfonate; PFC, perfluorinated compounds, *n* = 3. Patterns: NFH, No fluorescence on sperm head; AF, acrosome fluorescence; EHZ; equatorial head zone fluorescence; AE, acrosomal and equatorial fluorescence. ^a, b, c, d^ Different letters in same column represent significant statistical differences.
